# Data on pesticide exposure and mental health screening of family farmers in Brazil

**DOI:** 10.1016/j.dib.2019.103993

**Published:** 2019-05-24

**Authors:** Rafael Junqueira Buralli, Helena Ribeiro, Renata Spolti Leão, Rejane Correa Marques, Jean Remy Daveé Guimarães

**Affiliations:** a Departamento de Saúde Ambiental, Faculdade de Saúde Pública, Universidade de São Paulo, São Paulo, Brazil; b Centro de Tecnologia em Nanomateriais e Grafeno – CTNANO/UFMG, Belo Horizonte, Brazil; c Universidade Federal do Rio de Janeiro, Campus Macaé, Macaé, Brazil; d Instituto de Biofísica Carlos Chagas Filho, Universidade Federal do Rio de Janeiro, Rio de Janeiro, Brazil

**Keywords:** Farmers, Mental health, Occupational exposure, Pesticides, Agricultural worker's diseases

## Abstract

This dataset is part of a risk assessment project that evaluated the human health effects of pesticide exposure in São José de Ubá, State of Rio de Janeiro. This region is one of the greatest tomato producers in Brazil, and pest control is commonly based on the use of pesticides. We interviewed 78 smallholder family farmers about sociodemographic characteristics, pesticide use and exposure, assessed blood cholinesterase as biomarkers (*n* = 70), and screened all participants for probable common mental disorders through the Self-Reporting Questionnaire (SRQ-20).

**Table:** Specifications table

Subject area	*Environmental Health; Psychology; Public Health*
More specific subject area	*Exposure Assessment; Farmers; Mental Health; Pesticides*
Type of data	*Tables*
How data was acquired	*Smallholder family farmer's interviews, and blood cholinesterase tests.*
Data format	*Raw, filtered and analyzed*
Experimental factors	*We interviewed a sample of Brazilian smallholder family farmers to assess the sociodemographic characteristics, pesticide use and exposure, and common mental disorders through Self-Reporting Questionnaire (SRQ-20), and quantified acetylcholinesterase (AChE) and butyrylcholinesterase (BChE) activity in blood. This dataset include unpublished data on pesticide exposure and mental health effects among pesticide sprayers and current or former helpers involved in tomato cultivation in Brazil.*
Experimental features	*Data collection on sociodemographic characteristics, pesticide exposure and use, and screening of probable common mental disorders.*
Data source location	*Data collected in the municipality of São José de Ubá (-21.365600; -41.951715), located in the Northwest of the State of Rio de Janeiro, Brazil.*
Data accessibility	*Sociodemographic, lifestyle and exposure characteristics were partially presented in BURALLI* et al. *(2018), while complementary data on pesticide exposure, and unpublished mental health data are available in this brief.*
Related research article	*1) Buralli R.J., Ribeiro H., Mauad T., Amato-Lourenço L.F., Salge J.M., Diaz-Quijano F.A., Leão R.S., Marques R.C., Silva D.S., Guimarães J.R.D. Respiratory condition of family farmers exposed to pesticides in the state of Rio de Janeiro, Brazil. Int J Environ Res Public Health. 2018. 15, 1203.*

**Table:** 

**Value of the Data** • This brief provides unpublished data on occupational exposure to pesticides and prevalence of common mental disorders among Brazilian family farmers, divided by pesticide sprayers and current or former helpers; • This data can be used to compare sociodemographic and pesticide exposure characteristics, and cholinesterase activity in smallholder farmers cultivating tomato or other crops, and farmers performing different agricultural tasks in Brazil and elsewhere; • This data can be used to compare the prevalence of common mental disorders among individuals involved in different agricultural tasks, and non-exposed populations.

## 1 Data

This data was collected as part of the project “*Human health risk assessment by exposure to metals and pesticides in the municipality of São José de Ubá, State of Rio de Janeiro*”. Smallholder family farming for tomato cultivation is the main source of income in this region. During the crop season of 2014, 78 residents of the rural area of São José de Ubá (SJU) were interviewed about sociodemographic and lifestyle factors, pesticide use and exposure, and health effects. Based on their cultivation tasks, participants were divided into two groups: pesticide sprayers (*n* = 42), and current or former helpers (*n* = 36). Eight individuals refused to participate in the blood tests or their samples were insufficient for analysis, and acetylcholinesterase (AChE) and butyrylcholinesterase (BChE) activity were quantified in 70 individuals. This brief presents unpublished data on participant's exposure to pesticides, cholinesterase measures, mental health assessed through the Self-Reporting Questionnaire (SRQ-20), and the types of pesticides commonly used in SJU. This data must be interpreted with caution due to the small sample size. Participant's sociodemographic and lifestyle aspects, pesticide exposure and respiratory health within this population were discussed elsewhere [1]. Table 1 shows participant's characteristics such as sex, age, duration of work in agriculture, age when started working or helping in crop activities, body mass index, smoking habits, cholinesterase activity, and total of affirmative answers in the SRQ-20. All participants presented normal AChE, while BChE alteration are presented in Table 1. Table 2 presents the prevalence of affirmative answers in each question of the SRQ-20. This research protocol was approved by the University Hospital Clementino Fraga Filho of the Federal University of Rio de Janeiro (HUCFF/UFRJ; 30459814.5.0000.5257), and all participants provided written informed consent.

**Table 1:** Data on sociodemographic and pesticide exposure characteristics, and mental health screening among smallholder family farmers in São José de Ubá, Brazil, during the tomato crop season, 2014.

		Sex^a^	Age	Work years^b^	Start age^c^	BMI^d^	Smoking^e^	AChE^f^	BChE^f^	Altered BChE^g^	SRQ-20^h^
help1		M	74	66	8	27.0	0.0	–	–	–	8
help2		F	56	45	10	25.0	1.4	1.18	4.23	0	5
help3		M	62	48	14	23.4	33.0	0.97	2.02	1	3
help4		M	54	40	13	35.1	0.5	1.18	2.60	0	2
help5		F	36	28	8	28.4	0.0	1.49	2.85	0	0
help6		F	37	25	12	31.6	0.0	1.66	4.16	0	9
help7		F	41	24	17	26.8	3.3	2.00	4.80	0	17
help8		M	55	45	10	20.7	55.5	1.28	2.97	0	10
help9		M	59	41	14	23.2	0.0	1.42	5.27	0	7
help10		M	56	49	7	22.8	25.5	1.81	4.73	0	10
help11		F	75	40	15	19.9	0.0	–	–	–	9
help12		F	72	52	19	32.0	0.0	1.23	2.42	0	0
help13		F	39	25	14	39.6	0.0	1.76	4.11	0	3
help14		F	47	14	30	38.7	0.0	0.77	1.75	0	7
help15		F	41	15	15	27.7	0.0	1.85	3.49	0	4
help16		F	34	20	13	23.0	0.0	1.12	3.55	0	0
help17		F	65	46	9	28.3	0.0	–	–	–	15
help18		F	46	39	7	32.0	0.0	1.27	4.15	0	18
help19		F	63	20	23	25.8	2.0	0.87	3.30	0	0
help20		F	46	26	15	40.1	0.0	–	–	–	9
help21		F	66	25	36	42.0	15.0	1.14	2.67	0	15
help22		F	44	13	31	23.9	2.0	1.22	2.45	0	9
help23		F	23	6	17	37.4	0.0	1.68	5.26	0	4
help24		F	60	30	19	26.2	0.0	1.39	4.53	0	3
help25		F	38	23	10	29.4	0.0	1.08	4.16	0	0
help26		M	30	9	16	17.4	0.0	1.05	2.62	0	4
help27		F	33	6	25	41.0	0.0	1.26	3.49	0	2
help28		F	55	48	7	23.2	1.2	2.14	4.11	0	17
help29		F	44	10	26	23.5	0.0	0.94	4.00	0	9
help30		F	37	5	12	28.6	0.0	1.47	2.68	0	1
help31		F	41	5	31	27.6	0.1	–	–	–	4
help32		M	39	27	9	23.7	0.0	0.91	1.79	1	7
help33		F	67	33	34	24.4	0.0	1.97	4.34	0	3
help34		F	33	5	23	25.8	0.0	1.81	3.50	0	0
help35		M	23	3	12	25.7	0.0	1.13	4.06	0	4
help36		M	52	20	7	22.0	12.0	0.81	1.88	1	17
spra1		M	49	41	7	21.8	10.0	–	–	–	4
spra2		M	20	7	13	22.5	8.0	–	–	–	4
spra3		M	48	36	12	28.4	10.0	0.80	1.84	1	0
spra4		M	44	25	19	25.7	60.0	0.93	2.04	1	8
spra5		M	49	39	10	28.4	0.0	1.14	2.75	0	0
spra6		M	39	33	6	26.0	0.0	0.72	2.86	0	3
spra7		M	44	36	8	29.6	0.0	1.16	2.79	0	4
spra8		M	48	33	15	24.1	0.0	0.88	2.60	0	0
spra9		M	45	38	7	29.4	0.0	1.05	3.58	0	2
spra10		M	18	5	13	19.9	0.0	1.11	3.50	0	0
spra11		M	49	34	15	26.8	15.0	1.11	4.83	0	5
spra12		M	24	12	12	25.0	0.0	1.34	4.87	0	2
spra13		M	46	38	8	27.8	0.0	1.70	4.18	0	2
spra14		M	42	34	8	20.5	25.5	1.77	4.01	0	0
spra15		M	53	41	12	26.6	0.0	–	–	–	13
spra16		M	61	54	7	20.3	0.8	2.04	2.42	0	0
spra17		M	43	36	7	28.4	0.0	0.58	1.67	1	0
spra18		M	27	12	15	20.2	0.0	0.89	3.40	0	1
spra19		M	53	30	15	24.3	0.0	1.27	2.78	0	1
spra20		M	34	26	8	29.4	0.1	1.30	4.79	0	2
spra21		M	48	30	17	22.4	0.0	1.26	3.74	0	2
spra22		M	33	12	20	33.2	0.0	1.54	5.15	0	6
spra23		F	33	14	19	32.5	0.0	1.64	3.61	0	2
spra24		M	53	40	13	24.7	0.0	1.00	3.10	0	10
spra25		F	50	13	37	17.5	17.0	2.42	4.38	0	11
spra26		M	22	12	10	24.3	0.0	1.34	1.77	1	6
spra27		M	48	35	13	22.3	0.0	0.75	1.78	1	1
spra28		M	24	11	12	20.4	0.0	1.69	3.52	0	6
spra29		F	20	8	12	20.8	0.0	1.63	4.53	0	10
spra30		M	25	10	15	20.8	0.0	0.62	1.72	1	1
spra31		M	32	18	12	20.5	0.0	0.84	1.78	1	2
spra32		F	51	42	9	26.8	0.0	1.64	4.34	0	2
spra33		M	41	32	9	29.0	3.0	0.80	1.73	1	2
spra34		M	44	37	7	21.3	16.5	0.64	1.53	1	11
spra35		M	44	30	14	20.1	10.5	0.84	2.09	1	3
spra36		M	34	28	6	22.6	0.0	1.64	4.80	0	15
spra37		M	50	31	8	25.0	10.5	1.02	2.09	1	4
spra38		M	39	27	12	27.6	0.0	0.78	2.75	0	0
spra39		F	40	8	32	33.8	0.0	1.78	3.52	0	4
spra40		F	38	24	13	23.7	0.0	1.89	3.93	0	2
spra41		M	61	40	8	27.0	10.2	1.18	3.47	0	5
spra42		F	28	20	8	18.1	0.0	1.42	2.87	0	4

a Male = M and female = F.

b Years working or helping in crop activities.

c Age they started to work or helping in crop activities.

d Body mass index.

e Smoking habit in pack-years.

f Values expressed in mmol/min/mg.

g Normal = 0 and altered = 1, considering 0.56 mmol/min/mg for AChE in both genders, and 2.29 for men and 1.61 mmol/min/mg for BChE for women.

h Total of affirmative answers in SRQ-20.

**Table 2:** Prevalence of affirmative answers at the SRQ-20 among smallholder family farmers in São José de Ubá, Brazil, during the tomato crop-season, 2014.

SRQ-20 - Affirmative answers	Total % (*n* = 78)	Sprayers % (*n* = 42)	Current or former helpers % (*n* = 36)
Q1. Often have headaches	33.3 (26)	28.6 (12)	38.9 (14)
Q2. Poor appetite	15.4 (12)	9.5 (4)	22.2 (8)
Q3. Sleep badly	39.7 (31)	26.2 (11)	55.6 (20)
Q4. Easily frightened	41.0 (32)	33.3 (14)	50.0 (18)
Q5. Hands shake	23.1 (18)	21.4 (9)	25.0 (9)
Q6. Feel nervous, tense or worried	61.5 (48)	54.8 (23)	69.4 (25)
Q7. Poor digestion	9.0 (7)	2.4 (1)	16.7 (6)
Q8. Trouble thinking clearly	26.9 (21)	21.4 (9)	33.3 (12)
Q9. Feel unhappy	21.8 (17)	14.3 (6)	30.6 (11)
Q10. Cry more than usual	16.7 (13)	9.5 (4)	25.0 (9)
Q11. Difficult to enjoy your daily activities	17.9 (14)	11.9 (5)	25.0 (9)
Q12. Difficult to make decisions	25.6 (20)	21.4 (9)	30.6 (11)
Q13. Daily work suffering	28.2 (22)	38.1 (16)	16.7 (6)
Q14. Unable to play a useful part	15.4 (12)	7.1 (3)	25.0 (9)
Q15. Lost interest in things	17.9 (14)	11.9 (5)	25.0 (9)
Q16. Feel that you are a worthless person	19.2 (15)	4.8 (2)	36.1 (13)
Q17. Thought of ending your life	11.5 (9)	7.1 (3)	16.7 (6)
Q18. Feel tired all the time	26.9 (21)	19.0 (8)	36.1 (13)
Q19. Uncomfortable feelings in the stomach	24.4 (19)	21.4 (9)	27.8 (10)
Q20. Easily tired	30.8 (24)	16.7 (7)	47.2 (17)
SRQ above the standard cutoff *	33.3 (26)	23.8 (10)	44.4 (16)

* Standard cutoff level defined as ≥ 6 positive answers for men and ≥8 positive answers for women.

## 2 Experimental design, materials, and methods

This cross-sectional study was conducted in SJU, a small municipality from the State of Rio de Janeiro, Brazil, which has about 7000 inhabitants, mostly residents of the rural area (55%). In SJU, only 14.6% of the population is formally employed, and 40% have a per capita monthly income of about $125 US dollars, which is equivalent to less than 1/2 Brazilian minimum wage [2]. Small-scale family farming is the primary source of income in SJU, mainly producing tomato with the use of pesticides. SJU is one of the largest tomato producers in Brazil, and from 2007 to 2017, the average yearly production ranged from 21.000 to 32.000 tons [3]. During the tomato crop season of 2014 (July and August), upon SJU resident's and stakeholder's indication, we conveniently recruited and interviewed 82 smallholder family farmers older than 18 years old. Lately, four individuals who never helped in crop activities were excluded, and the final sample of this brief is 78 smallholder family farmers. Based on their self-declared cultivation tasks, participants were divided into: a) sprayers (*n* = 42), those daily involved in all crop activities, including pesticide handling and application; b) current or former helpers (*n* = 36), those who helped in crop activities except pesticide spraying.

### 2.1 Questionnaire assessments

Individual face-to-face interviews were conducted at participant's homes, neighborhood's schools and health units to collect sociodemographic, lifestyle, and pesticide exposure data. Interviews were based on a questionnaire adapted from the “Protocol for evaluation of chronic pesticide poisoning” of the Health Department of the State of Paraná, Brazil [4]. We collected sociodemographic and personal data such as age, BMI, income, smoking habit, and data on pesticide exposure, e.g. the age they started to help in crop activities and years of work, home exposure to pesticides, home distance to crop areas, use of personal protection equipment, and whether they had or not previous training and technical support. Sprayers were asked what pesticides they frequently use, and they were presented as extremely, highly, moderately or low toxic, according to the Brazilian National Sanitary Surveillance Agency – ANVISA [5]. Forty-nine pesticides from thirty-one chemical groups were cited, including organophosphate pesticides like *Acephate* and *Chlorpyrifos*, carbamates like *Mancozeb, Methomyl*, and *Cymoxanil*, pyrethroids like *Lambda-Cyhalothrin* and *Deltamethrin*, among others. Pesticides mentioned were classified as moderately (46%), extremely (30%) and highly toxic (20%) to humans [5]. Pesticides prohibited in Brazil for tomato cultivation (*Glyphosate*, *Paraquat, Chlorpyrifos,* and *2,4-D)*, and banned in Brazil *(Endosulfan)* were also cited [6].

The prevalence of CMD was assessed through the Self-Reporting Questionnaire (SRQ-20), proposed by the World Health Organization as a response to the lack of a low-cost and easy tool for psychiatric screening, recommended for community studies and basic care. The questionnaire has 20 binary questions (yes/no) about depressive and anxiety signs, reduced vital energy, and somatic symptoms. It was validated in Brazil with high sensitivity (83%) and specificity (80%), and the standard cutoff for probable CMD was set in six or more positive answers for men and eight or more for women [7].

### 2.2 Blood sampling and cholinesterase determination

Blood samples were collected by qualified personnel from 70 participants to measure acetylcholinesterase (AChE) and butyrylcholinesterase (BChE) activity, which may indicate the recent exposure to organophosphorus (OP) and carbamate (CM) pesticides. Analysis were conducted by the *Centro de Estudos da Saúde do Trabalhador e Ecologia Humana (CESTEH/FIOCRUZ)* through spectrophotometry (Shimadzu UV/VIS 1601) by using the Ellman method modified to use frozen samples [8], which is indicated when samples are collected far from the laboratory. Participant's cholinesterase activity was compared to the reference values proposed by CESTEH/FIOCRUZ based in studies with non-exposed individuals, which is 0.56 mmol/min/mg for AChE in both genders, and 2.29 for men and 1.61 mmol/min/mg for BChE for women. Values above these levels might be considered as normal.
