# Supplementary material for: Data on pesticide exposure and mental health screening of family farmers in Brazil
Source: Data Brief. 2019 May 24;25:103993. doi: 10.1016/j.dib.2019.103993 (PMC6595273; doi:10.1016/j.dib.2019.103993)
Supplement: Multimedia component 2 [file mmc2.docx]

**DIB-D-19-00726 – Label description**

Ident – Family farmer’s identity

expo_group – Exposure group: 0 helper; 1 sprayer

age – in years

sex – 1 male; 2 female

race – Declared race: 1 white; 2 black; 3 mixture (*“pardo”*)

marital – Marital status: 1 married or cohabiting partner; 2 single or divorced

fam_income – Monthly family income (in BR$): 1 up to 2 salaries; 2 more than 2 salaries

own_house – Own house: 0 no; 1 yes

crop_situation – Situation of work related to the crop: 0 currently not working or helping; 1 sharecropper (*“meeiro”,* those who plant with others and afterwards divide the products and profits); 2 own/family crop (those who cultivate in their own lands); 3 tenant/land renter (those who sponsor the cultivation process and earn a greater portion of the profits)

rural_debt – Have rural debts: 0 no; 1 yes

bmi – Body mass index (as continuous)

bmi_clas – Body mass index classes: 0 low or normal weight; 1 overweight; 2 obese

educ – Years of education (in years)

pack_years – Smoking habits (in pack-years)

smoke – Smoking status: 0 never; 1 former (ex-smoker); 2 current smoker

alcohol – Alcohol consumption: 0 no; 1 yes

wor_agric_years – Duration of work in agriculture (in years)

age_start – Age when started working/helping in the crop (in years)

age_dico – Age when started working/helping in the crop: 1 more than 16y; 2 up to 15y

home_expo – Have home exposure: 0 no; 1 yes

home_dist_crop – Home distance from crop areas: 0 more than 500m; 1 less than 500m

use_off_season – Pesticide use in the off-season: 0 no; 1 yes

ppe_any – Use of any personal protection equipment (PPE): 0 no; 1 yes

ppe_mask – Use of mask: 0 no; 1 yes

epi_visor – Use of visor: 0 no; 1 yes

epi_hat – Use of hat: 0 no; 1 yes

ppe_gloves – Use of gloves: 0 no; 1 yes

ppe_boots – Use of boots: 0 no; 1 yes

ppe_coverall – Use of coverall: 0 no; 1 yes

prev_poison – Have ever had previous poisoning: 0 no; 1 yes

pre_training – Have ever had previous training: 0 no; 1 yes

wash_hands – Commonly wash hands after working in crop: 0 no; 1 yes

shower_after – Commonly shower after working in crop: 0 no; 1 yes

consu_food_water – Commonly consumes food and water in crop areas: 0 no; 1 yes

ache_med – Acetylcholinesterase enzymes quantification (as continuous)

bche_med – Butyrylcholinesterase enzymes quantification (as continuous)

alt_bche – Butyrylcholinesterase below the reference value (2.29 mmol/min/mg for men, and 1.61 mmol/min/mg for BChE for women)

SRQ_q1 - SRQ-20 Question 1 – Often have headaches: 0 no; 1 yes

SRQ_q2 - SRQ-20 Question 2 – Have poor appetite: 0 no; 1 yes

SRQ_q3 - SRQ-20 Question 3 – Sleep badly: 0 no; 1 yes

SRQ_q4 - SRQ-20 Question 4 – Easily frightened: 0 no; 1 yes

SRQ_q5 - SRQ-20 Question 5 – Hands shake: 0 no; 1 yes

SRQ_q6 - SRQ-20 Question 6 – Feel nervous, tense or worried: 0 no; 1 yes

SRQ_q7 - SRQ-20 Question 7 – Have poor digestion: 0 no; 1 yes

SRQ_q8 - SRQ-20 Question 8 – Have trouble thinking: 0 no; 1 yes

SRQ_q9 - SRQ-20 Question 9 – Often feel unhappy: 0 no; 1 yes

SRQ_q10 - SRQ-20 Question 10 – Cry more than usual: 0 no; 1 yes

SRQ_q11 - SRQ-20 Question 11 – Have difficult to enjoy your daily activities: 0 no; 1 yes

SRQ_q12 - SRQ-20 Question 12 – Have difficult to make decisions: 0 no; 1 yes

SRQ_q13 - SRQ-20 Question 13 – Have daily work suffering: 0 no; 1 yes

SRQ_q14 - SRQ-20 Question 14 – Feel unable to play a useful part: 0 no; 1 yes

SRQ_q15 - SRQ-20 Question 15 – Lost interest in things: 0 no; 1 yes

SRQ_q16 - SRQ-20 Question 16 – Feel that you are a worthless person: 0 no; 1 yes

SRQ_q17 - SRQ-20 Question 17 – Ever thought of ending your life: 0 no; 1 yes

SRQ_q18 - SRQ-20 Question 18 – Feel tired all the time: 0 no; 1 yes

SRQ_q19 - SRQ-20 Question 19 – Feel uncomfortable feelings in the stomach: 0 no; 1 yes

SRQ_q20 - SRQ-20 Question 20 – Are easily tired: 0 no; 1 yes

SRQ_dico – SRQ above the cutoff level (men ≥ 6 women ≥ 8): 0 no; 1 yes
